# RNAseq analysis of *Cellvibrio japonicus* during starch utilization differentiates between genes encoding carbohydrate active enzymes controlled by substrate detection or growth rate

**DOI:** 10.1128/spectrum.02457-23

**Published:** 2023-10-06

**Authors:** Cecelia A. Garcia, Jeffrey G. Gardner

**Affiliations:** 1 Department of Biological Sciences, University of Maryland, Baltimore County, Baltimore, Maryland, USA; Forschungszentrum Jülich GmbH, Juelich, Germany

**Keywords:** Amylase, *Cellvibrio japonicus*, Glycoside Hydrolase, Maltose, Starch

## Abstract

Bacterial utilization of starch is increasingly of interest as the importance and contributions of animal gut microbiomes become more defined. Consequently, identifying and characterizing the bacterial enzymes responsible for the degradation, transport, and metabolism of starch will enable developments in pharmaceutical, biotechnological, and culinary industries searching for novel prebiotics, carrier molecules, and low glycemic index sweeteners. The current challenge is that bacteria proficient at starch utilization often have hundreds of carbohydrate active enzymes, and it is unclear which are essential for starch utilization using only homology-based bioinformatics or computational methods. Complementary experimental data are also needed, especially to understand the regulation of bacterial starch utilization. We have completed an RNAseq analysis of the Gram-negative bacterium *Cellvibrio japonicus* and found that it has sophisticated regulation that includes substrate sensing and growth rate components for genes that encode starch-degrading enzymes. Among the 22 genes predicted to encode starch-active enzymes, *C. japonicus* has 10 alpha-amylases, 4 alpha-glucosidases, 2 pullulnases, and 2 cyclomaltodextrin glucanotransferases, 15 of which were up-regulated during exponential growth on starch and 8 up-regulated in stationary phase. Growth analyses with an enzyme secretion deficient mutant of *C. japonicus* suggested that secreted amylases are essential for this bacterium to degrade starch. Our approach of coupling a physiological growth assay with transcriptomic data provides a platform to identify targets for further genetic or biochemical analysis that can be broadly applied to other starch-utilizing bacteria.

**IMPORTANCE:**

Understanding the bacterial metabolism of starch is important as this polysaccharide is a ubiquitous ingredient in foods, supplements, and medicines, all of which influence gut microbiome composition and health. Our RNAseq and growth data set provides a valuable resource to those who want to better understand the regulation of starch utilization in Gram-negative bacteria. These data are also useful as they provide an example of how to approach studying a starch-utilizing bacterium that has many putative amylases by coupling transcriptomic data with growth assays to overcome the potential challenges of functional redundancy. The RNAseq data can also be used as a part of larger meta-analyses to compare how *C. japonicus* regulates carbohydrate active enzymes, or how this bacterium compares to gut microbiome constituents in terms of starch utilization potential.

## OBSERVATION

Plant starches represent an abundant energy source for microbes able to degrade these complex polysaccharides. Bacteria possessing α-amylases, which are Glycoside Hydrolase family 13 (GH13) Carbohydrate Active enZymes (CAZymes), can degrade the amylose and amylopectin components of starch (1, 2). These enzymes are of particular interest for biotechnological and biomedical applications and may also contribute to a better understanding of how gut microbiota thrive in a crowded community (3, 4). While α-amylase enzymology is well described, there are less data available on how genes encoding amylases are regulated, particularly within bacteria that possess multiple starch-degrading enzymes. For example, the Gram-negative saprophyte *Cellvibrio japonicus* has 17 predicted genes that encode GH13 family enzymes (5). With this high amount of potential redundancy, it is unclear which *C. japonicus* amylases are essential versus those that provide more ancillary functions. Similar challenges can be found with other Gram-negative bacteria of medical importance that possess multiple genes that encode GH13 enzymes, including *Bacteroides thetaiotaomicron* (8 genes) and *Klebsiella oxytoca* (12 genes) (6, 7). To complement a recent genome announcement describing strains of *C. japonicus* with altered abilities to utilize α-diglucosides (8, 9), we performed a transcriptomic analysis of *C. japonicus* while utilizing starch. As a community resource, this data set can be used to help discover and subsequently characterize critical starch-specific CAZymes in related bacteria or used in meta-analyses of microbial communities.

### Secreted amylases are essential for starch utilizing by *C. japonicas*

**Fig 1:**
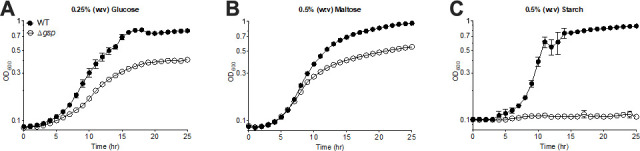
Growth analysis of *C. japonicus* strains. *C. japonicus* wild-type and a Δ*gsp* strain were grown in MOPS-defined minimal media containing either (**A**) 0.25% (w:v) glucose, (**B**) 0.5% (w:v) maltose, or (**C**) 0.5% (w:v) starch. Experiments were performed in biological triplicate with error bars representing standard deviation. All bacterial strains were grown at 30°C, liquid cultures were incubated with shaking (200 RPM) for aeration. Growth experiments were performed in 96-well microtiter plates with optical density at 600 nm (OD_600_) measured using an Epoch microplate spectrophotometer (BioTek).

We chose potato starch (Fisher; S516-100) as the experimental substrate because it is comprised of α−1,4 glycosidic bonds (with α−1,6 branches), which is a native substrate for GH13 CAZymes. We grew *C. japonicus* strains in MOPS-defined media (TekNova; M2106) with glucose, maltose, or starch as the sole carbon source (Fig. 1) prior to RNAseq analysis. We characterized growth of the wild-type strain in addition to a Type II Secretion System mutant (Δ*gsp*), which has been previously shown to be essential for secreting CAZymes in *C. japonicus* (10, 11). This strain has also proven insightful for what degradation systems require exported enzymes in *C. japonicus*, as it was previously shown that secreted CAZymes are unnecessary for unbranched xylan utilization (12). For this study, the wild-type strain grew well on each substrate, while the ∆*gsp* strain displayed a well-characterized aeration-dependent growth defect on both glucose and maltose (12 – 15). On starch, the Δ*gsp* strain was completely unable to grow. This strongly suggested that secreted amylases are essential for *C. japonicus* to utilize this substrate. A previous bioinformatic analysis found that 8 of the 17 predicted GH13 enzymes did not have a cleavable signal peptide (5); therefore, these CAZymes likely play a physiological role that is distinct from starch degradation. With nine amylases that are secreted in *C. japonicus*, growth data from a wild-type strain or genome sequence alone are insufficient to identify the amylase-encoding genes that are critical for starch utilization, which was the rational for the RNAseq.

### Transcriptomic analysis of *C. japonicus* during starch utilization identified CAZyme-encoding genes regulated by substrate or growth rate

We employed RNAseq to identify the CAZyme-encoding genes expressed during growth on starch in comparison to glucose and collected cell pellets for RNAseq during mid-exponential growth and early stationary phase as previously described (12, 14 – 17). Previously published RNAseq data for *C. japonicus* grown using glucose (GSE90955) were used for comparative analysis (14). The RNAseq data generated for *C. japonicus* on starch are available at NCBI GEO under Accession number GSE206866.

**Fig 2:**
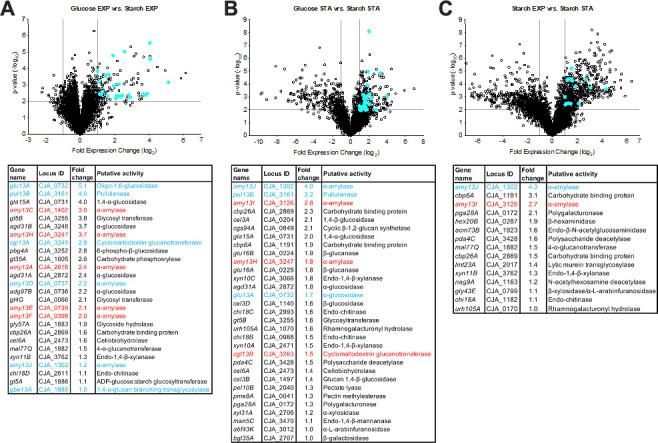
Differential gene expression of wild-type *C. japonicus* during exponential growth on starch versus glucose. Wild-type *C. japonicus* cultures were grown on media containing either glucose (0.25%, w:v) or starch (0.5%, w:v) and utilized for transcriptomic analysis. Briefly, biological triplicate samples were collected at the onset of exponential phase (0.1 > OD_600_ >0.2) and again during early stationary phase (OD_600_ >1.0). Metabolism was halted with a stop solution of ethanol and saturated phenol (vol:vol 19:1). Cells were pelleted by centrifugation (8,000 *g*, 4°C, 5 min), the supernatant decanted, and the cell pellet then flash frozen in a dry ice/ethanol bath for 5 min. Frozen cell pellets were then stored at −80°C. RNA extraction and QC analyses were performed by GeneWiz (South Plainfield, NJ, USA). All RNA samples were observed to have a RIN >8.0 and of sufficient quantity to proceed with RNAseq. Sequencing was performed at GeneWiz with an Illumina HiSeq2500 (50 bp single-end reads; >10 million reads/sample). The raw data were returned and then analyzed using tools accessed from the Galaxy platform, specifically FastQC, ENSEMBLE, HISAT2, and DESeq2, all with default settings (19). Gene expression values were quantile-normalized for all samples and log_2_-transformed before performing a Student’s *t*-test. Significantly expressed genes were defined as those with a *P*-value <0.01 and log_2_ fold change >2. Volcano plots of all *C. japonicus* genes were plotted as fold change (log_2_) and *P*-value (−log_10_) observed during exponential growth versus glucose (**A**), stationary phase versus glucose (**B**), or exponential versus stationary phase on starch (**C**). Each gene is represented by a black circle while up-regulated CAZyme-encoding genes are colored blue. Gray lines represent significance cut-off values of −log_10_(*P*-value) >2.0 and log_2_ (fold change) >1.0. In the corresponding data in tablular form, the GH13 genes are color coded, where blue denotes genes predicted to encode for cytoplasmic or surface-attached enzymes, and red indicates genes predicted to encode secreted enzymes.

When comparing gene expression of starch-grown cells versus glucose-grown cells during exponential growth, there were 135 genes that were significantly (*P* >0.01) up-regulated (fold change >2.0) that could be broadly grouped by gene ontology (GO) (18) as having roles in carbon metabolic processes (GO:0005975), glycogen biosynthesis (GO:0005978), and phosphorelay signal transduction (GO:0000160). Among the up-regulated genes were 26 CAZyme-encoding genes (Fig. 2), which included 7 predicted α-amylase-encoding genes, 6 alpha-glucosidase-encoding genes, 1 pullulnase-encoding gene, and 1 cyclomaltodextrin glucanotransferase-encoding gene. The remaining CAZyme-encoding genes had predicted roles in cellulose, xylan, and chitin degradation, which was interesting but expected as previous RNAseq studies found overlap among glycoside hydrolase-encoding genes that were up-regulated in *C. japonicus* when pure polysaccharide substrates were used (14, 15, 17). These previous reports argued that while the bacterium can specifically sense different polysaccharide substrates and up-regulate the appropriate CAZyme-encoding genes, unrelated CAZyme-encoding genes on the same regulatory circuits were also up-regulated. Given the complex nature of environmental polysaccharides, this strategy is likely to maximize potential substrate utilization.

For gene expression analysis of starch-grown *C. japonicus* cells versus glucose-grown cells during stationary phase, 289 genes were significantly up-regulated and could be grouped by the GO terms carbohydrate metabolic processes (GO:0005975), signal transduction (GO:0007165), chemotaxis (GO:0006935), and phosphorelay signal transduction (0000160). Among the up-regulated genes were 31 CAZyme-encoding genes, including 3 predicted α-amylase-encoding genes, 3 alpha-glucosidase-encoding genes, 1 pullulnase-encoding gene, and 1 cyclomaltodextrin glucanotransferase-encoding gene. Overall, the CAZyme-encoding genes up-regulated during stationary phase were previously shown to encode proteins possessing a wide array of carbohydrate-degrading activities (14, 15, 17). Specifically, RNAseq analyses of cellulose, chitin, or mannan utilization during stationary phase identified a diverse set of CAZyme-encoding genes, which argues for a system of shared regulators during polysaccharide degradation and consistent with previously transcriptomic studies in *C. japonicus* (12, 20).

Comparing gene expression analysis of starch-grown *C. japonicus* cells between exponential growth and stationary phase identified 628 significantly up-regulated genes that could be grouped by the GO terms chemotaxis (GO:0006935), bacterial-type flagellum assembly (GO:0044780), organization (GO:0044781), and motility (GO:0071973), signal transduction (GO:0000160 and GO:0007165), transcription regulation (GO:0006355), and carbohydrate metabolism (GO:0005975). There was a total of 15 CAZyme-encoding genes that were up-regulated, which included only two predicted α-amylase-encoding genes but no alpha-glucosidase-encoding genes, pullulnase-encoding genes, or cyclomaltodextrin glucanotransferase-encoding genes. As only two amylase genes (*amy13I; amy13J*) were regulated (but not exclusively) by growth rate, we hypothesize that the major regulatory mechanism that *C. japonicus* employs to control starch-degradative enzymes is through substrate detection. This mechanism is markedly different than some Gram-positive bacteria, which have been shown to exclusively control amylase-encoding genes, and more generally those involved in carbohydrate degradation, via growth rate or catabolite repression (21 – 23).

### Conclusion

We have reported here an RNAseq dataset for the Gram-negative saprophyte *C. japonicus* during starch utilization that can serve as resource for future meta-analyses of polysaccharide degradation and metabolism in bacteria. Given the problems associated with defining physiological function based solely on bioinformatic predictions (24), integrating transcriptomic/proteomic, genetic, and/or physiological data provides much needed insight required to identify among a collection of related enzymes those that are truly important for the utilization of a given substrate. It may be of particular use for comparative studies of Gram-negative bacteria found in gut microbiomes, in agriculturally important microbes, and/or biotechnology applications to narrow down target enzymes. In this last case, the RNAseq data can be used to refine target lists given the potential redundancy found in *C. japonicus* and related bacteria due to the abundance of genes that encode carbohydrate active enzymes.
